# Pulmonary Artery Remodelling Assessed by Four-Dimensional Flow Magnetic Resonance Imaging in Pulmonary Arterial Hypertension and Atrial Septal Defect

**DOI:** 10.1016/j.cjcpc.2025.03.004

**Published:** 2025-03-19

**Authors:** Estibaliz Valdeolmillos, Emmanuelle Fournier, Hichem Sakhi, Paul Vignaud, Marion Audié, Marc-Antoine Isorni, Bastien Provost, Florence Lecerf, Clément Batteux, Grégoire Albenque, Olivier Sitbon, David Montani, Xavier Jais, Laurent Savale, Marc Humbert, Arshid Azarine, Sébastien Hascoët

**Affiliations:** a Department of Congenital Heart Diseases, Reference Center for Complex Congenital Disease M3C, Rare Disease Ntework, Hôpital Marie Lannelongue, Le Plessis-Robinson, France; b Paris-Saclay University, INSERM UMR_S 999, Hypertension Pulmonaire: Physiopathologie et Innovation Thérapeutique (HPPIT), AP-HP, Hôpital Bicêtre, Hôpital Marie Lannelongue, ERN-LUNG, Le Plessis-Robinson, France; c Department of Cardiology, Hôpital Marie Lannelongue, Le Plessis-Robinson, France; d Department of Radiology, Les Hôpitaux Paris Saint-Joseph et Marie Lannelongue, Le Plessis-Robinson, France; e Research and Innovation Department, Marie Lannelongue Hospital, Le Plessis-Robinson, France; f Department of Respiratory and Intensive Care Medicine, Reference Center for Pulmonary Hypertension, Hôpital Bicêtre, Le Kremlin-Bicêtre, France

## Abstract

**Background:**

Pulmonary artery (PA) stiffness plays a significant role on right ventricular (RV) function, as evidenced in pulmonary arterial hypertension (PAH). In patients with atrial septal defect–related PAH (ASD-PAH), prognosis is dependent on RV function. We aimed to characterize PA remodelling and its relationship with RV function using 4-dimensional flow magnetic resonance imaging (4D flow MRI) in patients with ASD-PAH.

**Methods:**

We prospectively included 24 adults with ASD-PAH. They underwent 4D flow MRI and right heart catheterization within 24-48 hours. Remodelling of PA was analysed (stiffness, compliance, distensibility, elastance, and pulsatility) and PA vortex was evaluated. RV adaptation was measured by RV ejection fraction and RV-PA coupling.

**Results:**

The median age was 50 [41-55] years, and the median mean PA pressure and pulmonary vascular resistance were 50 [39-58] mm Hg and 8.0 [6.8-11.3] WU, respectively. Fourteen patients (58.3%) had RV dysfunction (RV ejection fraction <45%). All presented PA dilation (median main PA diameter: 42 [37-48] mm) and an increase in PA stiffness (10 [7.4-15.3]), whereas compliance and distensibility were decreased (2.1 [1.8-4.1] mm^2^/mm Hg and 0.19 [0.13-0.29] %/mm Hg). No correlation was found between PA dilation and indexes of PA remodelling. PA remodelling parameters were more severely impaired in patients with preserved RV function, with an increased stiffness (*P* = 0.01) and a decreased compliance and distensibility (*P* = 0.02).

**Conclusions:**

PA stiffness parameters characterized by 4D flow MRI were impaired in the population with ASD-PAH, and PA remodelling appears to be influenced by volumetric overload from the left-to-right shunt through the ASD.

## Résumé

**Contexte:**

La rigidité de l’artère pulmonaire joue un rôle significatif dans la fonction ventriculaire droite, comme en témoigne l’hypertension artérielle pulmonaire (HAP). Chez les patients atteints d’HAP liée à une communication interauriculaire (HAP-CIA), le pronostic dépend de la fonction ventriculaire droite. Nous avons cherché à caractériser le remodelage de l’artère pulmonaire et sa relation avec la fonction ventriculaire droite à l’aide d’un examen d’imagerie par résonance magnétique (IRM) de flux en quatre dimensions (4D) chez des patients atteints d’HAP-CIA.

**Méthodologie:**

Vingt-quatre adultes atteints d’HAP-CIA ont été inclus à l’étude de manière prospective. Ils s’étaient prêtés à un examen d’IRM de flux 4D et à un cathétérisme du cœur droit dans les 24 à 48 heures. Nous avons analysé le remodelage de l’artère pulmonaire (rigidité, compliance, capacité de dilatation, élastance et pulsatilité) et évalué le vortex dans l’artère pulmonaire. L’adaptation du ventricule droit a été mesurée à l’aide de la fraction d’éjection ventriculaire droite et du couplage ventricule droit-artère pulmonaire (VD-AP).

**Résultats:**

L’âge médian était de 50 ans [41 à 55]. La pression moyenne médiane dans l’artère pulmonaire et la résistance vasculaire pulmonaire étaient de 50 mmHg [39 à 58] et de 8,0 unités Wood [6,8 à 11,3], respectivement. Quatorze patients (58,3 %) présentaient une dysfonction ventriculaire droite (fraction d’éjection ventriculaire droite < 45 %). Tous présentaient une dilatation de l’artère pulmonaire (diamètre médian du tronc principal de l’artère pulmonaire : 42 mm [37 à 48]) et une augmentation de la rigidité de l’artère pulmonaire (10 [7,4 à 15,3]), tandis que la compliance et la capacité de dilatation étaient réduites (2,1 mm^2^/mmHg [1,8 à 4,1] et 0,19 %/mmHg [0,13 à 0,29], respectivement). Aucune corrélation n’a été observée entre la dilatation de l’artère pulmonaire et les indices du remodelage de l’artère pulmonaire. Les paramètres de remodelage de l’artère pulmonaire étaient plus fortement altérés chez les patients dont la fonction ventriculaire droite était préservée, en présence d’une rigidité accrue (*p* = 0,01) et en présence d’une compliance et d’une capacité de dilatation réduites (*p* = 0,02).

**Conclusions:**

Les paramètres de la rigidité de l’artère pulmonaire évalués à l’aide d’une IRM de flux 4D sont altérés dans la population atteinte d’HAP-CIA, et le remodelage de l’artère pulmonaire semble être influencé par la surcharge volémique associée au shunt gauche-droite dû à la CIA.

Atrial septal defects (ASD) represent the second most common congenital heart defect, with pulmonary arterial hypertension (PAH) occurring in up to 10% of cases.^1^ PAH is characterized by the remodelling of the pulmonary vascular bed, leading to an abnormal increase in pulmonary vascular resistance (PVR), which results in progressive right ventricular (RV) failure,^2^ representing the most common cause of morbidity and mortality in PAH.^3^^,^^4^ The mechanisms by which isolated pretricuspid shunts can cause severe PAH are debated. In ASD, the increase in pulmonary blood flow is believed to cause right heart volume overload, resulting in progressive dilation of RV and pulmonary artery (PA). The RV function and its adaptation to increase preload and afterload (RV-PA coupling) are crucial in this population influencing symptomatology and outcomes.^5^ PA elasticity plays a crucial role in RV-PA coupling, and PA stiffness can be assessed by right heart catheterization (RHC) and magnetic resonance imaging (MRI). PA stiffness has previously been shown to reflect disease severity and poor clinical outcomes in heterogeneous adult population with PAH.^6^, ^7^, ^8^ Alterations in the elastic properties of the PA assessed by MRI and their association with RV dysfunction have been documented in PAH, suggesting that PA stiffness is a major contributor to RV dysfunction.^9^, ^10^, ^11^, ^12^ Furthermore, 4-dimensional flow MRI (4D flow MRI) analysis of the appearance and duration of blood vortex in the PAs has been correlated with PAH severity.^13^ However, the PA remodelling and its relationship with RV function in this specific population with ASD-PAH remain to be fully characterized. The objective of this study was to characterize PA remodelling using 4D flow MRI and to provide insights about its relationship with RV function in patients with ASD-PAH.

## Materials and Methods

### Study design

We conducted a prospective study at Marie Lannelongue Hospital, a tertiary centre for complex congenital heart diseases (*M3C network* *rare diseases*), and surgical management of PAH, in collaboration with Kremlin-Bicetre Hospital, French reference centre for PAH (*PulmoTension network*). Between June 2018 and June 2021, a total of 24 consecutive patients aged ≥18 years diagnosed with PAH^4^ (mean PA pressure (mPAP) >20 mm Hg, PVR >2 WU, PA wedge pressure ≤15 mm Hg) associated with ASD (isolated or associated with partial anomalous pulmonary venous return) were enrolled. Patients underwent 4D flow MRI and RHC within 24-48 hours. Clinical, echocardiographic, and biological data were collected at the same time. The institutional review board (IRB) with appropriate consent to supply data (IRB number: IRB 2018-A00332-53) approved the study. All patients gave their informed consent. This study complies with the STARD guidelines^14^ and was registered on ClinicalTrial.gov (NCT03928002).

### Cardiac magnetic resonance protocol

4D flow MRI was performed in a 1.5-Tesla system (Optima MR 450 W; General Electric Healthcare, Waukesha, WI) with a 32-channel body coil.

#### Ventricular volumes and function

**Figure 1:**
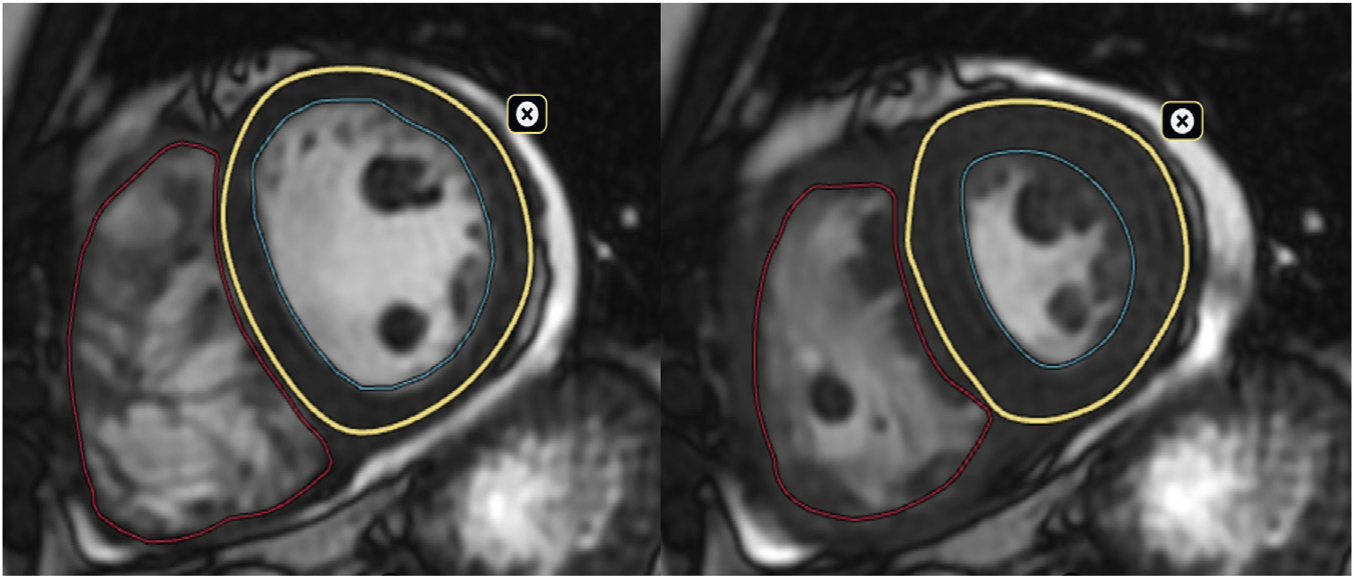
Right ventricular and left ventricular end-diastolic and end-systolic volumes and ejection fraction were measured by manually contouring epicardial (**yellow line**) and endocardial (**blue** and **red lines**) borders from short-axis using Arterys software.

Balanced steady-state free precession cine images were captured in both short-axis and long-axis orientations for assessing the volumes and function of the left ventricle and RV. RV and left ventricle end-diastolic, end-systolic indexed volumes (EDVi and ESVi) and ejection fraction (EF) were measured by manually contouring epicardial and endocardial borders from short-axis images (Fig. 1). RV dysfunction was defined as RVEF <45%. RV dilatation was defined as RVEDVi >112 mL/m^2^ for women and >121 mL/m^2^ for men, and RVESVi >52 mL/m^2^ for women and >59 mL/m^2^ for men.^15^ RV-PA coupling was quantified as the ratio of PA effective elastance (Ea, index of arterial load) to RV maximal end-systolic elastance (Emax, index of contractility) according to the simplified formula: Ea/Emax = RVESV/RVSV (RVESV: RV end-systolic volume, RVSV: RV stroke volume).^16^

#### PA evaluation

**Figure 2:**
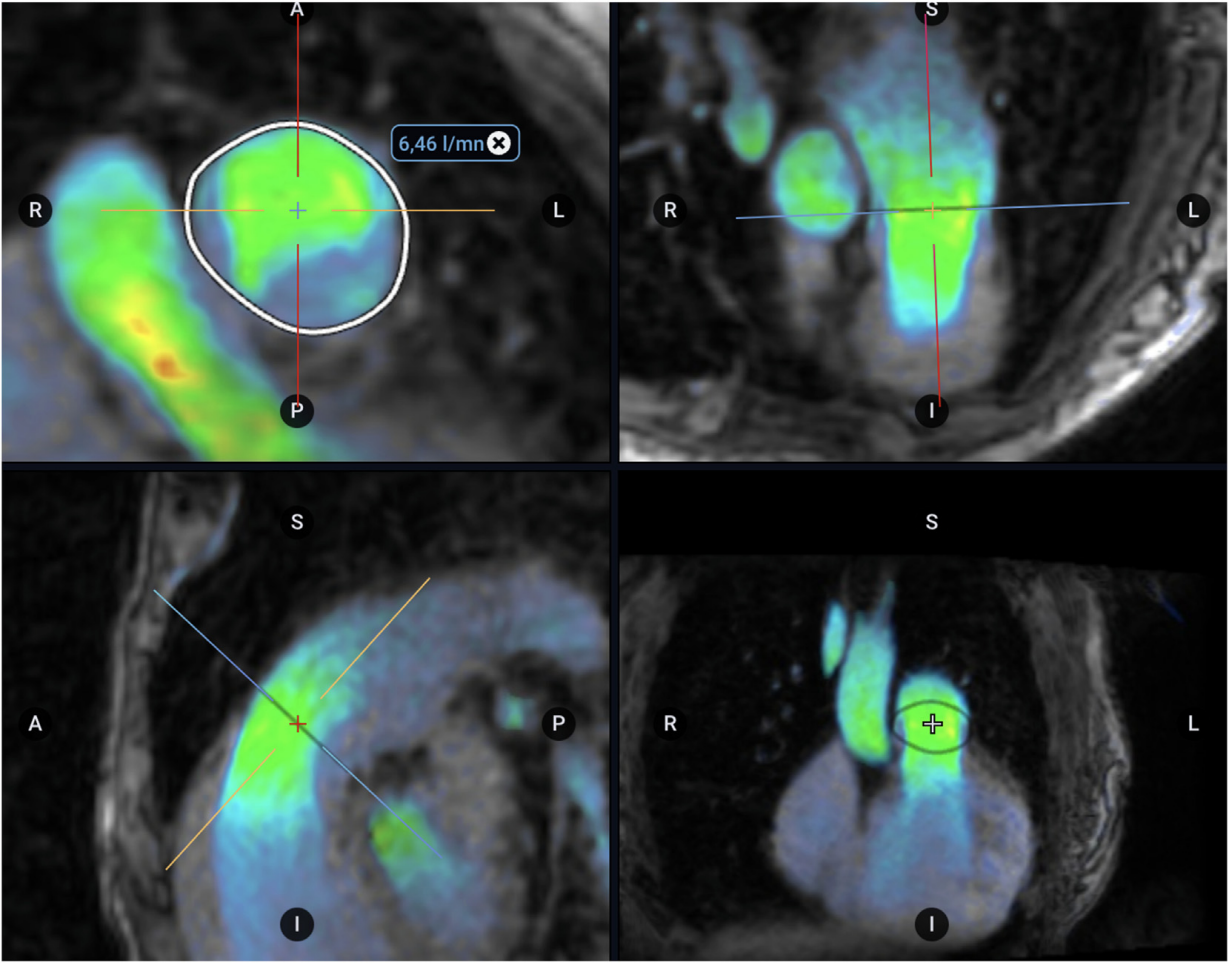
Pulmonary artery (PA) area measured by contouring the PA wall in systole and diastole in a plane perpendicular to the main PA using Arterys software.

2D-phase-contrast imaging was performed in PA and ascending aorta to measure cross-sectional areas and average blood flow. The PA area was measured by contouring the PA wall in systole and diastole in a perpendicular plane to the main PA (MPA), right PA (RPA), and left PA (Fig. 2). Anterograde, retrograde, and net flows (difference between anterograde and retrograde flow) were measured on 2D imaging to determine the measurement of Qp/Qs.^17^ The PA diameter was measured in systole. Most studies consider a normal MPA size of <28.9 mm for men and <26.9 mm for women or the upper limit of 29-30 mm without distinguishing between genders.^18^, ^19^, ^20^ According to previous studies, dilated MPA was considered when the diameter was ≥30 mm, significantly dilated when the MPA diameter was ≥40 mm and extremely dilated when the MPA diameter was ≥50 mm.

The elastic properties of PA were analysed with the following parameters:^6^^,^^21^^,^^22^

• Stiffness index β was defined as the slope of the function between arterial pressure and arterial distension: Stiffness index β = (Ln(systolic PAP/diastolic PAP))/((PA max Surface – PA min Surface)/PA min Surface).
• Compliance was defined as an absolute change in lumen area for a given change in pressure: PA compliance (mm^2^/mm Hg) = (PA max Surface – PA min Surface)/PP (PP: pulsatile pulmonary arterial pressure measured at RHC).
• Distensibility was defined as the relative change in lumen area for a given change in pressure: AP distensibility (%/mm Hg) = (PA max Surface – PA min Surface)/(PP × PA min Surface) × 100.
• Elastance (also called elastic modulus) was defined as the change in pressure resulting a relative increase in lumen area: PA elastance (mm Hg) = PP × PA min Surface/(PA max Surface – PA min Surface) (PP: pulse pressure).
• Pulsatility (also called relative area change) was defined as the relative variation of the vessel lumen during the cardiac cycle: PA pulsatility (%) = (PA max Surface – PA min Surface)/PA min Surface × 100. Relative area change represents solely a vascular geometric property.

#### 4D flow acquisition and analysis

The 4D flow sequences were performed on DV26 using a Cartesian sampling (Kat-ARC) acceleration factor of 6, with retrospective electrocardiogram gating and multiNEX respiratory compensation. To cover the whole thoracic area, a coronal 4D flow sequence was acquired, after intravenous injection of a bolus of 0.1 mmol/kg of gadobutrol (Gadovist Bayer, Berlin, Germany), followed by a slow perfusion of another 0.1 mmol/kg of gadobutrol. We established the velocity encoding in a range of 200-300 cm/s, assessing 30 cardiac frames for every cardiac cycle. This led to a reconstructed spatial resolution of 1.4-1.8 × 1.4-1.8 × 1.1-1.4 mm^3^. The flip angle was 12°, whereas the echo time and repetition time were 2.2 ms and 4.1 ms, respectively.

**Figure 3:**
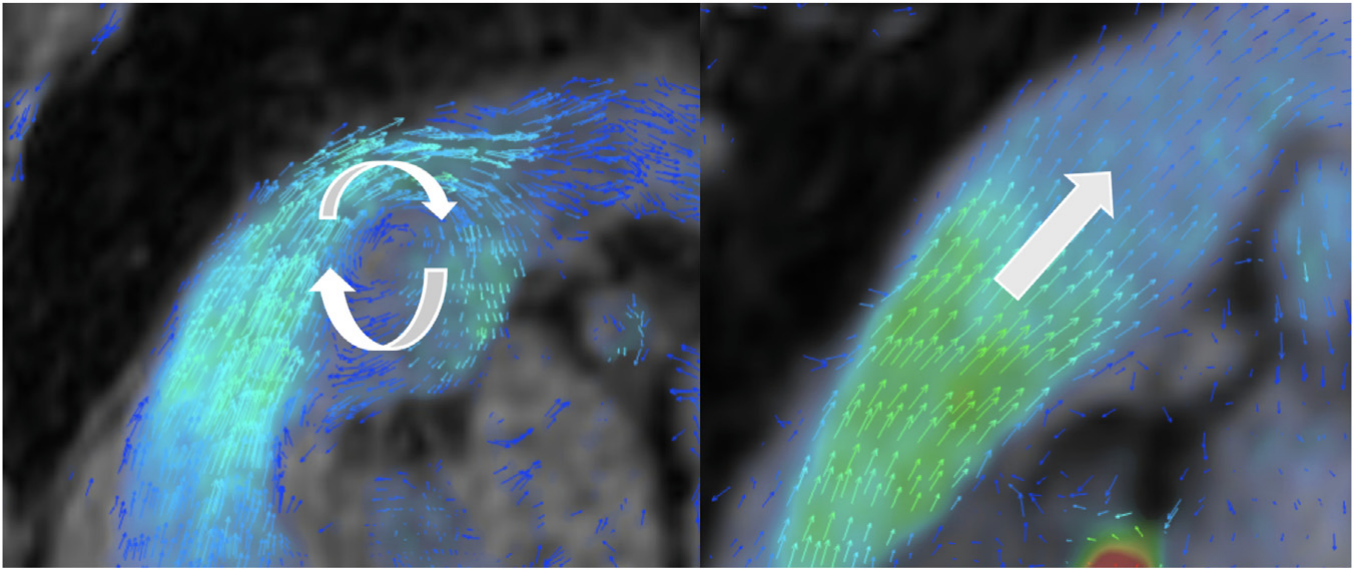
Laminar (**left**) and vortical (**right**) flow within the pulmonary artery using 4D flow acquisition on Arterys software.

Analysis of the 4D flow images was performed using Tempus Pixel (Chicago, IL) after semiautomatic calibration (static tissue detection, phase offset, and eddy current correction) helped by deep learning. Orthogonal planes were carefully placed on the MPA and the aorta, enabling the measurement of both pulmonary and systemic flows. This method allowed for the calculation of the shunt fraction. Vortical flows in PA were visually analysed. A vortical blood flow was characterized as a closed concentric ring-shaped flow, and its rotational axis was determined to be perpendicular to the PA through visual analysis, in accordance with prior descriptions (Fig. 3).^13^ Its proportionate duration in relation to the entire cardiac cycle (%) was recorded.

### Right heart catheterization

RHC was performed in haemodynamically stable and spontaneously breathing patients, under local anaesthesia, without oxygen support, allowing direct measurement of oxygen consumption, as previously described.^23^ Qp and Qs were calculated using the direct Fick principle. Haemodynamic measurements included right atrial pressure; left atrial pressure; PA wedge pressure; mean, systolic, and diastolic PAP; superior vena cava oxygen saturation; peripheral arterial oxygen saturation; and systolic aortic pressures. PVR was calculated as the transpulmonary pressure gradient/Qp ratio.

### Statistical analysis

Continuous variables with normal distribution (confirmed by a Shapiro-Wilk test) were expressed as mean ± standard deviation. Data that did not show a normal distribution are expressed as median and quartiles (first and third). Qualitative variables are expressed as a percentage with their number. To determine a significant difference in the case of a normal distribution of quantitative data, *t* tests or analysis of variance was used to compare 2 groups or more, and in the case of data not normally distributed, the Mann-Whitney *U* test or Kruskal-Wallis tests were used where appropriate. To compare categorical, discrete, or binary data, a Pearson χ^2^ test was used. To analyse the impact of risk factors on RVEF, RV-PA coupling, and N-terminal pro–B-type natriuretic peptide values, univariate linear regressions were performed. When multiple variables had a significant mean difference, a forward stepwise algorithm was conducted to select the most impactful one(s) in a final model. The significance level was set at 5%; consequently, the 95% confidence intervals were calculated. All data processing, statistical analyses, and graphs were performed using R (version 4.3.2; R Foundation for Statistical Computing, Vienna, Austria), with the help of the “ggplot2” and “StepReg” packages.

## Results

### Study population

Baseline characteristics are summarized in Table 1. Four transplantations were reported (3 double-lung transplantations and 1 heart-lung transplantation). Two patients died while awaiting transplant.

**Table 1:** Baseline and haemodynamic characteristics according to right ventricular function with 4-dimensional flow magnetic resonance imaging

Characteristics	Total (N = 24)	RVEF ≥45% (N = 10)	RVEF <45% (N = 14)	*P* value
Age (y)	50 [41-55.25]	51 [48-55.75]	42 [40.25-53.75]	0.25
Female	21 (87.5)	10 (100)	11 (78.6)	0.24
Body mass index (kg/m^2^)	22 [21-26]	24 [21-27]	22 [21-23]	0.44
Age at PAH diagnosis	37 [30-45]	34 [26-43]	39 [31-47]	0.43
Age at ASD diagnosis	35 [27-43]	26.5 [22.5-36.5]	39 [31-47]	**0.05**
Type of ASD				1
ASD OS	18 (75)	8 (80)	10 (71)	
ASD OS + APVR	3 (12.5)	1 (10)	2 (14)	
ASD sinus venosus	3 (12.5)	1 (10)	2 (14)	
Type of PAH				1
Left-to-right shunt	11 (46)	6 (60)	5 (36)	
Eisenmenger	13 (54)	4 (40)	9 (64)	
Saturation (%)	92 [86-95]	94.5 [92-97]	90 [86-95]	0.07
PAH therapy				0.31
Single therapy	5 (21)	1 (10)	4 (29)	
Dual therapy	12 (50)	7 (70)	5 (36)	
Triple therapy	7 (29)	2 (20)	5 (36)	
NYHA class				1
II	13 (54)	5 (50)	8 (57)	
III	11 (46)	5 (50)	6 (43)	
Sign RV failure	3 (12.5)	0 (0)	3 (21)	0.24
6 MWD (m)	402 [349-458]	456 [402-490]	379 [331-401]	**0.02**
Clinical risk scores				
Reveal 2.0				**0.05**
Low risk	12 (50)	8 (80)	4 (29)	
Intermediate risk	5 (21)	1 (10)	4 (29)	
High risk	7 (29)	1 (10)	6 (43)	
ESC/ERS score				**0.01**
Low risk	11 (46)	8 (80)	3 (21)	
Intermediate risk	1 (4)	1 (10)	1 (7)	
High risk	12 (50)	1 (10)	10 (72)	
nt-proBNP (pg/mL)	404 [279-816]	302 [190-425]	776 [322-1562]	**0.01**
Creatinin (mmol/L)	70 [65-77]	75 [66-77]	69 [60-74]	0.34
RAP (mm Hg)	6 [3.8-8.3]	6.5 [4-10]	5.5 [3.3-6.8]	0.18
PVR (WU)	8.0 [6.8-11.3]	7.9 [4.4-11.2]	8.3 [7.3-11.3]	0.83
mPAP (mm Hg)	50 [39-58]	53.5 [43-59]	46.5 [38.25-56.75]	0.52
Qp/Qs	1.5 [1.0-1.9]	1.8 [1.4-2.4]	1.2 [0.7-1.5]	**0.02**
O_2_ rate in aorta (%)	93 [86-95]	94 [92-96]	88 [83-94]	**0.02**

All data are expressed with n (%) and median [Q1-Q3]. *P* = 0.05 or <0.05 indicates a statistically significant difference between these 2 groups and are indicated in bold.

4D flow MRI, four-dimensional flow magnetic resonance imaging; 6MWD, 6-minute walk test distance; APVR, anomalous pulmonary venous return; ASD, atrial septal defect; ERS, European Respiratory Society; ESC, European Society of Cardiology; mPAP, mean pulmonary arterial pressure; NYHA, New York Heart Association; nt-proBNP, N-terminal pro–B-type natriuretic peptide; OS, ostium secundum; PAH, pulmonary arterial hypertension; PVR, pulmonary vascular resistance; RAP, right atrial pressure; RV, right ventricle; RVEF, RV ejection fraction; WU, Wood’s units.

### PA characteristics

#### PA diameter

All patients presented MPA dilation (MPA diameter ≥30 mm) with a median MPA diameter of 42 [37-48] mm. Significant dilation (MPA diameter 40-49 mm) was present in 11 patients (46%), and extreme dilation (MPA diameter ≥50 mm) was present in 5 patients (21%). No significant difference was identified between PA dilatation and RV function.

#### PA remodelling parameters

All PA remodelling parameters were successfully measured in each patient. PA stiffness and elastance were increased, whereas compliance and distensibility were decreased in all patients compared with values in healthy patients, as shown in Table 2.^22^ RPA stiffness and elastance were significantly increased in normal RV function compared with RV dysfunction (25 [16-50] vs 12 [7.6-18], *P* = 0.01, and 1304 [720-2939] mm Hg vs 438 [348-1157] mm Hg, *P* = 0.02, respectively). A similar trend was observed for MPA and left PA but without reaching significance (Table 2). RPA stiffness was slightly positively correlated with RVEF (*r*^2^ = 0.22, *P* = 0.01). Compliance and distensibility were significantly decreased in patients with normal RV function compared with patients with RV dysfunction (0.77 [0.30-0.99] mm^2^/mm Hg vs 1.93 [0.77-2.64] mm^2^/mm Hg, *P* = 0.02, and 0.08 [0.03-0.14] %/mm Hg vs 0.23 [0.09-0.29] %/mm Hg, *P* = 0.02). No significant difference was observed between Eisenmneger syndrome or persistent left-to-right shunt concerning PA remodelling parameters, but a trend towards higher stiffness was noted in the left-to-right shunt group (Table 3).

**Table 2:** Four-dimensional flow magnetic resonance imaging characteristics according to right ventricular function

Characteristic	Population (N = 24)	RVEF ≥45% (N = 10)	RVEF <45% (N = 14)	*P* value
Cine				
RV-PA coupling	1.1 [0.6-1.7]	0.5 [0.5-0.6]	1.7 [1.2-1.9]	**<0.001**
RVEDVi (mL/m^2^)	136 [110-196]	112 [107-129]	182 [132-223]	**0.01**
RVESVi (mL/m^2^)	56 [51-68]	53 [48-61]	59 [53-75]	0.15
RVSV (mL)	24 [16-29]	25 [23-37]	18 [14-29]	0.22
Right atrial area (cm^2^)	23 [21-29]	22 [20-25]	23 [21-36]	0.33
RV mass index (g/m^2^)	66 [53-75]	55 [45-65]	72 [64-88]	**0.02**
LVEF (%)	59 [57-66]	65 [59-69]	58 [54-61]	**0.03**
Phase contrast				
MPA				
MPA maximum diameter (mm)	42 [37-48]	44 [38-47]	42 [38-51]	1
MPA maximum area (cm^2^)	14 [11-18]	16 [12-17]	14 [11-21]	1
MPA stiffness index β	10 [7.4-15.3]	13.5 [9.7-21.4]	8.6 [6.6-10.7]	0.15
MPA compliance (mm^2^/mm Hg)	2.1 [1.8-4.1]	1.9 [1.2-2.5]	2.4 [1.9-5.9]	0.11
MPA distensibility (%/mm Hg)	0.19 [0.13-0.29]	0.14 [0.08-0.24]	0.24 [0.15-0.29]	0.19
MPA elastance (mm Hg)	554 [341-775]	739 [450-1244]	421 [341-652]	0.19
MPA pulsatility index (%)	9.9 [7.6-14.1]	8.5 [6.1-12.6]	11.4 [8.2-14.8]	0.40
RPA				
RPA maximum diameter (mm)	34 [31-37]	32 [30-36]	36 [32-38]	0.43
RPA maximum area (cm^2^)	9.3 [7.6-11]	8.2 [7.1-10.4]	9.9 [7.8-11.2]	0.43
RPA stiffness index β	15.8 [8.6-25.2]	25.3 [16.1-50.1]	12.2 [7.6-17.7]	**0.01**
RPA compliance (mm^2^/mm Hg)	0.9 [0.7-2.1]	0.8 [0.3-1]	1.9 [0.8-2.6]	**0.02**
RPA distensibility (%/mm Hg)	0.13 [0.07-0.25]	0.08 [0.03-0.14]	0.23 [0.09-0.29]	**0.02**
RPA elastance (mm Hg)	810 [393-1345]	1304 [720-2939]	438 [348-1157]	**0.02**
RPA pulsatility index (%)	6 [4.6-11.6]	4.9 [2.4-7.5]	8.7 [5.5-13.3]	0.07
LPA				
LPA maximum diameter (mm)	31 [29-34]	31 [30-32]	33 [28-35]	0.66
LPA maximum area (cm^2^)	8.3 [7.7-9.7]	8.2 [8.1 -8.6]	8.9 [6.6-10.9]	0.58
LPA stiffness index β	13.9 [9.3-24.5]	18.4 [9.9-36.1]	13.7 [9.3-17.6]	0.31
LPA compliance (mm^2^/mm Hg)	1.1 [0.6-1.9]	0.8 [0.4-1.9]	1.3 [0.9-1.9]	0.26
LPA distensibility (%/mm Hg)	0.15 [0.07-0.28]	0.11 [0.06-0.21]	0.18 [0.08-0.28]	0.19
LPA elastance (mm Hg)	657 [361-1374]	1029 [479 -1675]	557 [358-1228]	0.19
LPA pulsatility index (%)	7.2 [5.1-13.2]	7.4 [3.8-12.5]	7.2 [5.3-12.9]	0.67
4D flow				
MAP net flow (mL/batt)	59 [44-74]	70 [52-92]	55 [42-69]	0.10
RPA net flow (mL/batt)	44 [36.9-56.3]	47.2 [37.5-55.8]	42.9 [37.6-55.2]	0.48
LPA net flow (mL/batt)	27.6 [23.6-38.5]	29.9 [25.3-52.1]	27.1 [21.5-36.7]	0.44
Qp/Qs	1.2 [0.9-1.7]	1.5 [1.1-1.8]	1.1 [0.8-1.6]	0.28
Vortex duration (%)	80 [63-87]	85 [80-87]	68 [58-85]	0.22

All data are expressed with median [Q1-Q3]. *P* = 0.05 or <0.05 indicates a statistically significant difference between these 2 groups and are indicated in bold.

LPA, left pulmonary artery; LVEF, left ventricular ejection fraction; MPA, main pulmonary artery; RPA, right pulmonary artery; RV, right ventricle; RVEDVi, RV end-diastolic indexed volume; RVEF, RV ejection fraction; RVESVi, RV end-systolic indexed volume; RV-PA, right ventricle-pulmonary artery; RVSV, right ventricular stroke volume.

**Table 3:** Four-dimensional flow magnetic resonance imaging characteristics comparing patients with Eisenmenger syndrome and those with persistent left-to-right shunt

Characteristics	Eisenmenger (N = 13)	Left-to-right shunt (N = 11)	*P* value
Cine			
RV-PA coupling	1.12 [0.56-1.87]	1.07 [0.56-1.69]	0.73
RVEDVi (mL/m^2^)	168 [129.7-208]	110 [108-138.15]	0.07
RVESVi (mL/m^2^)	56.0 [52.6-72]	56.4 [49.25-64.62]	0.77
RVSV (mL)	17.1 [13.1-24.3]	32.6 [27.2-39.4]	**0.002**
RA area (cm^2^)	25 [21-32]	21 [19-23]	0.08
RV mass index (g/m^2^)	65 [54-78]	67 [53.5-71.5]	0.77
LVEF (%)	58 [55-65.3]	59.8 [58-66.3]	0.38
Phase contrast			
MPA			
MPA max diameter (mm)	4.16 [3.53-5.31]	4.36 [3.97-4.72]	0.84
MPA max area (cm^2^)	13.6 [9.8-22.1]	14.9 [12.4-17.5]	0.84
MPA stiffness index β	9.2 [7.4-12.3]	13.9 [8-20.1]	0.39
MPA compliance (mm^2^/mm Hg)	2.06 [1.88-3.23]	1.88 [1.5-3]	0.30
MPA distensibility (%/mm Hg)	0.23 [0.14-0.29]	0.14 [0.09-0.26]	0.30
MPA elastance (mm Hg)	429 [341-732]	691 [390-1116]	0.30
MPA pulsatility index (%)	10.1 [9-13.5]	8.1 [6-15]	0.69
RPA			
RPA maximum diameter (mm)	34 [29-39]	35 [32-37]	1.00
RPA maximum area (cm^2^)	9 [6.7-11.9]	9.6 [7.9-10.5]	1.00
RPA stiffness index β	11.1 [7.5-16.8]	19.8 [15.7-27]	0.08
RPA compliance (mm^2^/mm Hg)	0.97 [0.53-2.10]	0.77 [0.63-1.33]	0.13
RPA distensibility (%/mm Hg)	0.21 [0.09-0.29]	0.08 [0.07-0.16]	0.13
RPA elastance (mm Hg)	482 [346-1071]	1185 [714-1387]	0.13
RPA pulsatility index (%)	10.8 [5.3-15.1]	5.6 [4.5-6.4]	0.15
LPA			
LPA maximum diameter (mm)	30 [28-36]	32 [31-34]	0.49
LPA maximum area (cm^2^)	8.2 [6.5-11.2]	8.6 [8.1-9.7]	0.62
LPA stiffness index β	10.1 [7.6-25.6]	15.6 [11.8-23.4]	0.28
LPA compliance (mm^2^/mm Hg)	1.46 [0.45-1.96]	1.01 [0.64-1.47]	0.28
LPA distensibility (%/mm Hg)	0.25 [0.07-0.29]	0.14 [0.08-0.18]	0.36
LPA elastance (mm Hg)	396 [341-1489]	729 [542-1312]	0.36
LPA pulsatility index (%)	11.1 [4.7-14]	6.7 [5.3-9.7]	0.53
4D flow			
RPA net flow (mL/batt)	44.8 [37.2-58]	43.3 [38.6-48.9]	0.61
LPA net flow (mL/batt)	25.6 [19.2-32.9]	31.9 [27.1-38.6]	0.19
Qp/Qs	1.1 [0.81-1.5]	1.41 [1.04-1.73]	0.37
Vortex duration (%)	80 [55-87]	80 [65.5-86.67]	0.86

All data are expressed with median [Q1-Q3]. *P* = 0.05 or <0.05 indicates a statistically significant difference between these 2 groups and are indicated in bold.

LPA, left pulmonary artery; LVEF, left ventricular ejection fraction; MPA, main pulmonary artery; RPA, right pulmonary artery; RV, right ventricle; RVEDVi, RV-end-diastolic indexed volume; RVEF, RV ejection fraction; RVESVi, RV-end-systolic indexed volume; RV-PA, right ventricle-pulmonary artery; RVSV, right ventricular stroke volume.

#### 4D flow parameters

4D flow parameters were similar in the groups with and without RV dysfunction (Table 2) and between patients with Eisenmenger syndrome and patients with persistent left-to-right shunt (Table 3). An increase in vortex duration was associated with a significant decrease in RV-PA coupling (*r*^2^ = 0.22, *P* = 0.01).

### RV function

Baseline and haemodynamic characteristics according to RV function are summarized in Table 1. RVEF was inversely correlated with RV-PA coupling (*r* = –0.91, *P* < 0.001). Patients with RV dysfunction had a significantly dilated RV (median RVEDVi 182 [132-223] mL/m^2^, *P* = 0.01), and a decreased median LVEF at 58% [53%-60%], *P* = 0.03, without clinical significance (Table 2). Patients with Eisenmenger syndrome demonstrated a significantly higher clinical risk score (Reveal 2.0 and European Society of Cardiology/European Respiratory Society risk score) (*P* = 0.05 and *P* = 0.04, respectively). RVEF was lower in the Eisenmenger group but without significance compared with the persistent left-to-right shunt group (35% [27%-52%] vs. 48% [33%-52%], *P* = 0.284). PVR and mPAP were not significantly different between the groups (Table 3).

## Discussion

To our knowledge, this is the first study to demonstrate the reliability of analysing PA stiffness parameters and flow pattern with 4D flow MRI in a rare condition of patients with ASD-PAH. We successfully conducted a multiparametric assessment of the elastic properties of the PA in all patients, including those with significantly dilated PA (21%). These findings provide valuable insights into the pathophysiological mechanisms underlying ASD-PAH, which may differ from other types of PAH.

The pathophysiology of PAH involves an increase in PVR, leading to increased stiffness and PA dilation, ultimately resulting in RV dysfunction.^2^ Conversely, patients with ASD experience a chronic right heart volume overload from birth, which contributes to progressive dilation of the RV and PA. This distinctive mechanism sets ASD-PAH apart from other PAH causes and even from post-tricuspid lesions.^24^ Moreover, the majority of patients remain asymptomatic for extended periods, leading to late diagnoses that often coincide with the onset of PAH. It is unclear whether the PAH is a consequence of increased blood flow through the left-to-right shunt, resulting in shear stress, or whether it is a result of a primary pulmonary vascular abnormality, or a combination of both.^25^ Genetic factors are likely to play a role in the development of pulmonary vascular disease.^26^, ^27^, ^28^

Advanced imaging techniques such 4D flow MRI offer a non-invasive method for evaluating PA morphology, flow, and phenotype, including the analysis of stiffness.^29^ Non-invasive assessment of PA stiffness has been previously documented in healthy patients and in heterogeneous populations with pulmonary hypertension.^6^^,^^22^ Our study found significantly increased PA stiffness, reduced PA compliance, and decreased distensibility compared with healthy individuals and even patients with other forms of pulmonary hypertension without congenital heart disease,^6^^,^^22^ indicating pronounced vascular remodelling specific to this population. Nevertheless, PA remodelling was not influenced by PA dilation, suggesting that the evolutive pattern of PA remodelling may differ in PAH-ASD.

Some studies on patients with PAH suggested a deleterious influence of increased PA stiffness on RV performance.^9^^,^^12^ Nevertheless, in our cohort, patients with normal RV function exhibited a tendency towards elevated PA stiffness, along with lower compliance and distensibility, compared with those with RV dysfunction. In addition, these patients were more likely to have a left-to-right shunt and a significantly higher Qp/Qs (*P* = 0.02). The cohort was insufficiently sized to corroborate discrepancies between patients with Eisenmenger syndrome and those with a persistent left-to-right shunt in PA remodelling parameters. However, a tendency towards elevated stiffness was noted in the left-to-right shunt group. These findings suggest that in patients with PAH-ASD, pulmonary remodelling is influenced not only by pressure overload from elevated PVR, as seen in other PAH types, but also by volumetric overload from the left-to-right shunt through the ASD. These two mechanisms interact synergistically, leading to distinct vascular remodelling patterns. As a result, PA stiffness may be more pronounced in patients with preserved RV function due to the additional stress caused by volume overload.

Concerning clinical status, patients with Eisenmenger syndrome were more severe, with higher clinical risk score, higher N-terminal pro–B-type natriuretic peptide values, and a lower 6-minute walk test distance with a trend of RV dysfunction as previously described.^30^ Outcomes analysis was constrained by the limited sample size. Three transplantations were reported, and 2 patients with Eisenmenger syndrome died while awaiting transplant.

4D flow MRI analysis of the appearance and duration of blood vortex in the PA arteries has been correlated with PAH severity and inversely related to RV-PA coupling.^13^ In our cohort, longer vortex duration was negatively associated with RV-PA coupling (*P* = 0.01), suggesting its potential as a marker for disease progression.^31^

Future research should explore how different degrees of PA stiffness abnormalities and vortex duration impact disease progression and treatment strategies.

### Limitations

Our study has several limitations, primarily related to the limited sample size, which may introduce selection bias, affect the robustness of our statistical analyses, and limit the generalizability of our findings to other clinical settings. However, because ASD-PAH is rare, our cohort is the largest to date specifically studying this homogeneous population using 4D flow MRI. In addition, our study was not age- or gender-matched, despite these being known factors influencing PA stiffness indices. The absence of a control group of patients with ASD without PAH limits our ability to fully assess the potential role of ASD alone in pulmonary stiffness. Lastly, due to the small number of adverse events in our cohort, it was not possible to establish associations with clinical outcomes. We considered RVEF measured by MRI as the primary endpoint, recognizing it as the gold standard; however, manual contouring may introduce biases and affect measurement reproducibility. Finally, our findings regarding the associations between RVEF and PA stiffness need to be confirmed in larger cohorts.

## Conclusion

PA remodelling in patients with ASD-PAH can be assessed by 4D flow MRI, which is a reliable tool. Patients with ASD-PAH exhibit significantly increased dilation and stiffness of PA, whereas compliance and distensibility were decreased. PA remodelling appears to be influenced by volumetric overload from the left-right shunt through the ASD, in addition to barometric overload.
